# Iatrogenic esophageal perforation in extremely preterm newborn with multiple comorbidities: case report and ethical considerations

**DOI:** 10.3389/fped.2026.1826347

**Published:** 2026-05-29

**Authors:** Gregorio Serra, Veronica Notarbartolo, Maria Rita Di Pace, Calogero Fabio Giardina, Valeria Guarneri, Ingrid Anne Mandy Schierz, Marco Pensabene, Maria Sergio, Mario Giuffre, Giovanni Corsello

**Affiliations:** 1 Neonatal Intensive Care Unit, Department for Health Promotion, Mother and Child Care, Internal Medicine and Medical Specialties “G. D'Alessandro” - University of Palermo, Palermo, Italy; 2 Pediatric Surgery Unit, Department for Health Promotion, Mother and Child Care, Internal Medicine and Medical Specialties “G.D'Alessandro” - Univerity of Palermo, Palermo, Italy; 3 Neonatal Intensive Care Unit, “Villa Sofia—V. Cervello” Joint Hospital, Palermo, Italy

**Keywords:** bedside ultrasonography, ethical decision-making, extremely low birth weight (ELBW), fatal outcome, iatrogenic injury, neonatal esophageal rupture

## Abstract

Neonatal esophageal perforation (EP) is a rare but potentially life-threatening complication, particularly in extremely low birth weight (ELBW) infants exposed to repeated invasive procedures. Although conservative management has progressively improved survival rates, mortality remains significant among the most vulnerable neonates. Literature studies predominantly describe favorable outcomes, possibly underrepresenting early lethal cases and overlooking the ethical implications of iatrogenic injury in infants at the threshold of viability. We report a fatal case of iatrogenic EP in an ELBW preterm infant and discuss diagnostic challenges, management strategies, and ethical considerations. A male infant was born at 27 weeks' gestation with severe intrauterine growth restriction and birth weight of 480 g. His clinical course was complicated by respiratory distress syndrome requiring prolonged mechanical ventilation, sepsis, patent ductus arteriosus, evolving bronchopulmonary dysplasia, pulmonary hypertension, hypertrophic cardiomyopathy, and periventricular leukomalacia. On day 24 of life, acute abdominal distension and clinical deterioration prompted radiographic and ultrasonographic evaluation. Imaging demonstrated malposition of the orogastric tube and right-sided pneumothorax, and subsequent mediastinal emphysema, confirming esophageal perforation. Conservative management was initiated, consisting of tube removal, *nil per os*, parenteral nutrition, broad-spectrum antibiotics, antifungal prophylaxis, respiratory and hemodynamic support, and pleural drainage. Despite timely recognition and adherence to recommended management strategies, the infant progressed to refractory multiorgan failure and died at one month of age. This case highlights the potentially fatal impact of EP in ELBW infants, in whom outcomes may be driven more by extreme prematurity and systemic instability than by the perforation itself. Bedside ultrasonography proved valuable for early detection and monitoring while limiting radiation exposure. Beyond clinical management, EP in critically ill preterm neonates raises complex ethical issues regarding proportionality of care, risk–benefit balance of invasive procedures, and transparent communication with families. Reporting severe and fatal cases is essential to improve awareness, refine preventive strategies, and foster ethically grounded decision-making in neonatal intensive care.

## Introduction

Neonatal esophageal perforation is an uncommon but potentially life-threatening complication, particularly in extremely low birth weight (ELBW) infants requiring prolonged intensive care and repeated invasive procedures (1, 2). Although advances in neonatal care have shifted management toward conservative strategies with improving survival rates, mortality remains significant in the most vulnerable populations (3–7). Furthermore, studies of the literature often describe favorable outcomes, potentially underrepresenting early lethal cases and the complex ethical implications associated with iatrogenic injury in infants at the threshold of viability (8). In this context, we present a fatal case of iatrogenic esophageal perforation in an ELBW preterm neonate, highlighting diagnostic challenges, clinical decision-making, and ethical considerations. In addition, we compare our experience with available literature to provide clinically and ethically relevant insights for practitioners involved in neonatal intensive care.

## Case presentation

**Figure 1:**
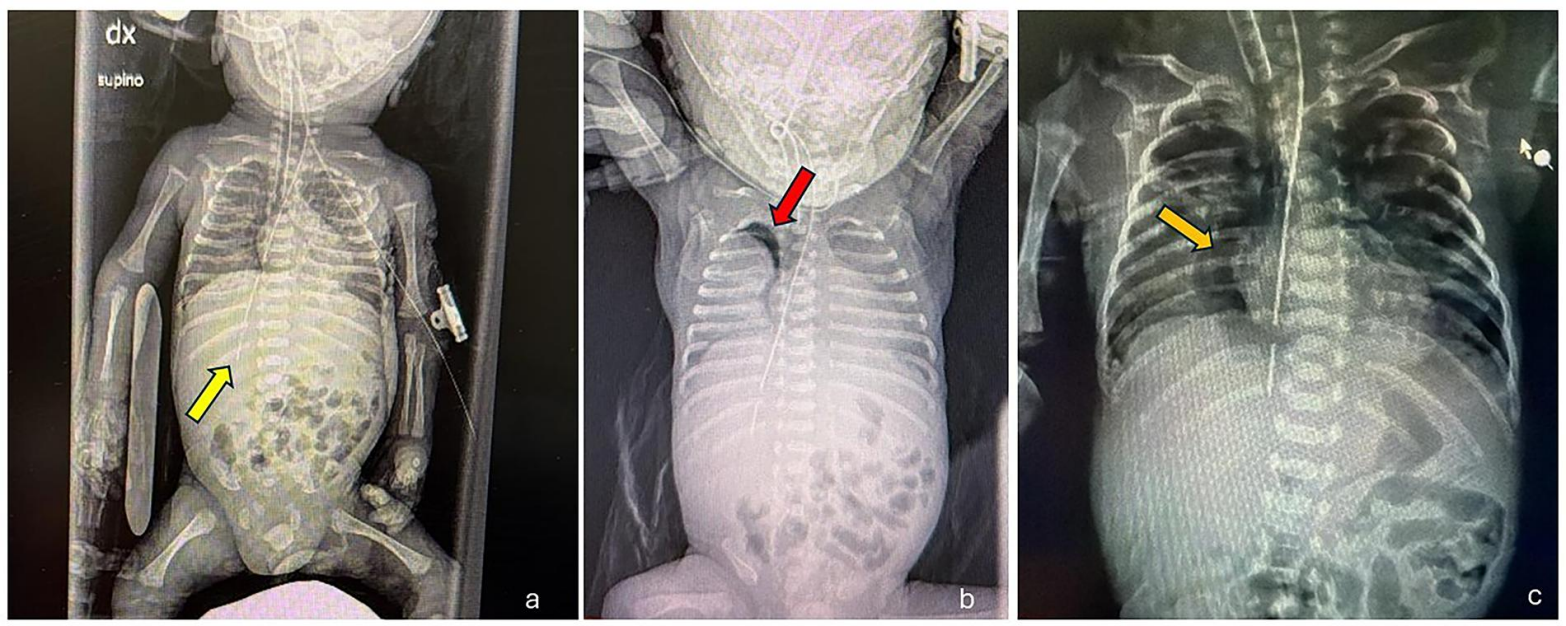
**(a)** Abnormal position of the nasogastric tube, with its tip located in the right hypochondrium at the level of the twelfth rib (yellow arrow). **(b)** In the upright image, a right-sided pneumothorax is visible, with partial lung collapse. The thickness of the air pocket indicated by the red arrow measures 5 mm. **(c)** Pneumothorax collection located in the right paramediastinal region (orange arrow) following esophageal perforation.

**Figure 2:**
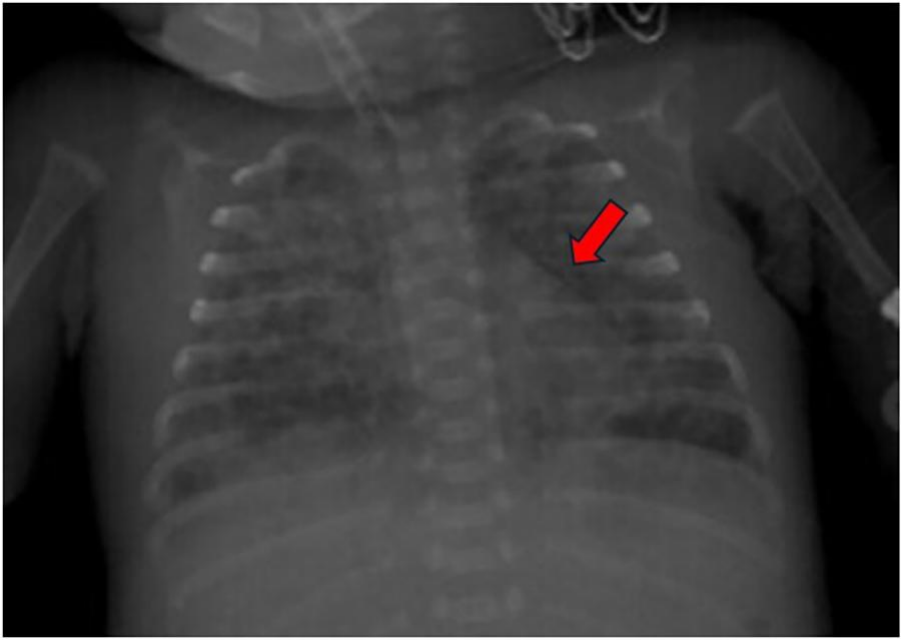
Reduction of the previously detected pneumothorax. A thin radiolucent layer persists in the left paracardiac region (indicated by the red arrow). The re-expanded lung shows diffuse and marked reticulonodular thickening with alternating multiple cyst-like formations, consistent with severe bronchopulmonary dysplasia (BPD).

**Figure 3:**
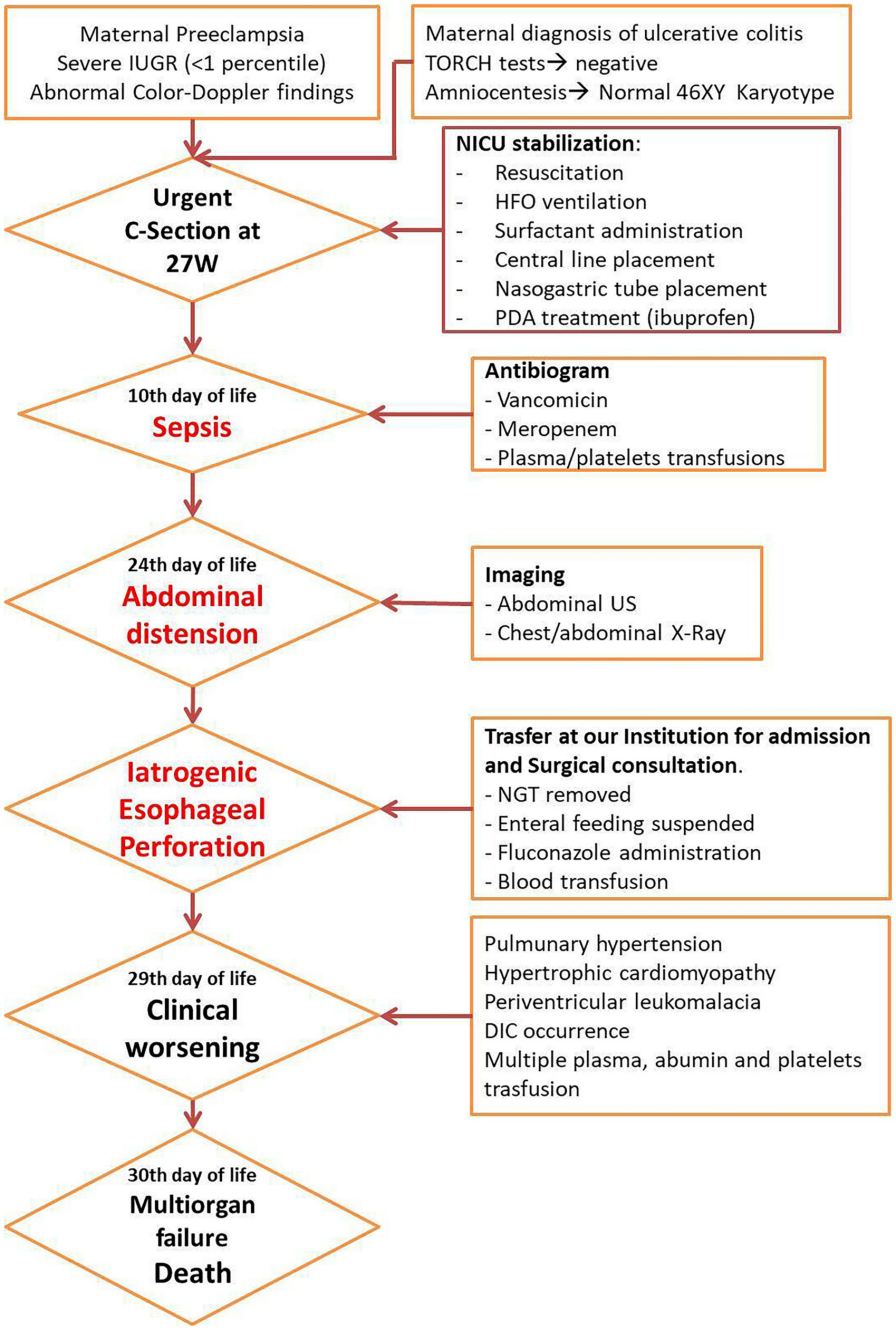
The flowchart resumes the clinical course of the reported case. Main clinical/therapeutical events are described.

A male preterm neonate was born at 27 weeks of gestation to non-consanguineous parents via emergency cesarean section due to maternal preeclampsia, severe early-onset intrauterine growth restriction (IUGR), and abnormal Doppler findings (absent A-wave in the ductus venosus and reversed flow in the umbilical arteries). The mother had a history of ulcerative rectocolitis, previously managed surgically without ongoing pharmacological treatment. Serial serological testing for TORCH infections was negative throughout the pregnancy. Prenatal screening indicated an increased risk for trisomies 13 and 21; however, amniocentesis confirmed a normal male karyotype (46, XY). Fetal biometry—including estimated fetal weight, head circumference, abdominal circumference, and femur length—was below the 1st percentile for gestational age (9), and fetal movements were notably reduced. Due to preterm premature rupture of membranes, the mother received intrapartum antibiotic prophylaxis, as well as steroid treatment preventing respiratory distress syndrome. The neonate's Apgar scores were 1, 5, and 8 at 1, 5, and 10 min, respectively. The infant weighed 480 g (2nd percentile, −2.15 SD), measured 28 cm in length (0th percentile, −2.83 SD), and had a head circumference of 21 cm (1st percentile, −2.5 SD), according to the Italian INeS Growth Charts (10). Immediate cardiopulmonary resuscitation (CPR) and endotracheal intubation were performed, followed by surfactant administration and initiation of high-frequency oscillatory ventilation (HFOV). During the Neonatal Intensive Care Unit (NICU) stay, parenteral nutrition was provided initially via an umbilical venous catheter during the first days of life and was subsequently continued through a left upper limb percutaneous epicutaneo-caval catheter, ensuring uninterrupted nutritional support. Minimal enteral feeding with mother's milk was also initiated within the first 72 h via nasogastric tube, at 10–24 mL/kg/day. On day 10 of life, sepsis caused by *Staphylococcus warneri* occurred and was treated with vancomycin and meropenem according to the antibiogram. Concomitantly, the neonate received ibuprofen for patent ductus arteriosus (PDA) closure and granulocyte colony-stimulating factor (G-CSF) to address neutropenia and support antimicrobial therapy. Multiple plasma and platelet transfusions were administered to manage coagulopathy and thrombocytopenia. Radiographic and ultrasonographic investigations, including chest x-rays and heart US, were performed also during the second week and at the beginning of the third week of life, in particular to monitor ongoing sepsis and PDA closure. On day 24 of life, the onset of abdominal distension and rigidity prompted chest and abdominal ultrasonography (US), which revealed malposition of the orogastric tube (OGT). This finding was subsequently confirmed by chest and abdominal radiographs (Figures 1a,b), demonstrating the tube tip just below the diaphragm, within the right hypochondrium at the level of the twelfth rib, and it was associated with difficulty in repositioning the OGT. In addition, a right anterior pneumothorax (PNX) was detected, requiring drainage (Figure 1c). A surgical consultation was requested, raising suspicion of iatrogenic esophageal perforation. Consequently, the patient was transferred to the NICU of our Institution, where a specialized Neonatal Surgery Unit is available, for further diagnostic assessment and management. After admission, the patient was critical. He was receiving invasive mechanical ventilation, initially via Synchronized Intermittent Positive Pressure Ventilation (SIPPV), followed by cycles of HFOV. Physical examination revealed a distended and tense abdomen and diffuse cutaneous pallor. Balanopreputial hypospadias was noted, although no dysmorphic features were observed. Cranial US showed no evidence of germinal matrix or intraventricular hemorrhage, with flowmetry and resistive indices within normal limits for gestational age. Given the evidence of esophageal perforation confirmed by x-Rays and US examinations, the reduction in the previously observed PNX along with signs of mediastinal emphysema (Figure 2), the nasogastric tube was removed, and no further insertion attempts were performed. Instead, a short oral tube, not ranging the esophagus, was positioned solely to allow superficial oropharyngeal suctioning of secretions. In light of multiple risk factors for invasive fungal infection, including extreme prematurity, prolonged broad-spectrum antibiotic therapy, and central venous catheterization, fluconazole prophylaxis was initiated in accordance with neonatal clinical practice recommendations (11). Furthermore, in the setting of persistent sepsis and the development of abdominal signs suggestive of possible gastrointestinal involvement, metronidazole was added to the antimicrobial regimen (i.e., vancomycin and meropenem) to ensure adequate anaerobic coverage. Hematochemical analysis revealed anemia [Hb 8.8 g/dL; transfusion threshold of 9 g/dL recommended for infants at ≥3 postnatal weeks receiving mechanical ventilation, according to the 2024 JAMA guidelines, (12)], hyponatremia (125 mEq/L, normal range 129–148 mEq/L), hypokalemia (3.6 mEq/L, normal range 4.5–7.1 mEq/L), and metabolic acidosis [pH 6.91, normal range 7.25–7.45; HCO3- 14 mmol/L, normal range 18–24 ± 2 mmol/L; base excess (BE) −10.9 mmol/L, normal range −5 to +2 mmol/L; lactate 10.7 mmol/L, normal range 1–3.5 mmol/L]. Thus, red blood cell transfusions, appropriate electrolyte supplementation, and bicarbonate correction were performed. Progressive systemic hypotension (50/24 mmHg; normal systolic blood pressure 55–75 mmHg and diastolic blood pressure 35–45 mmHg), and oliguria (urinary output 0.8 mL/kg/h over 24 h; normal urinary output ≥1 mL/kg/h over 24 h) (13), necessitated escalation of inotropic support. Approximately one week after the echocardiographic evaluation conducted at the referring center, which demonstrated mild left ventricular hypertrophy, moderate mitral insufficiency, and early signs of hypertrophic cardiomyopathy (HCM), the follow-up echocardiogram performed at our center soon after admission revealed closure of the ductus arteriosus, interventricular septum thickening (0.42 cm), mild left ventricular hypertrophy, pulmonary hypertension, and mediastinal emphysema. The initial decision by the referring center to close the PDA was likely prompted by evidence of significant left ventricular volume overload and associated hemodynamic burden. These findings suggest a pathophysiological link between the hemodynamically significant PDA and the progression of HCM, leading the ventricular myocardium to undergo compensatory hypertrophy, as reflected by interventricular septum thickening and mild left ventricular hypertrophy observed on echocardiography. This interpretation is further supported by a negative family history for cardiomyopathy, making a primary (genetic) HCM less likely. In this context, closure of the PDA with ibuprofen was deemed clinically justified to reduce left ventricular volume overload and limit further progression of HCM. Pulmonary hypertension, with a systolic pulmonary artery pressure (sPAP) of 63 mmHg (normal <35–40 mmHg) (14), was treated with a 250 mg/kg intravenous bolus of magnesium sulfate. Moreover, repeat cranial US demonstrated a 4 mm hyperechoic parenchymal area with a surrounding hypoechoic rim in the right frontal region, consistent with a subacute hemorrhagic suffusion. Additionally, marked hyperechogenicity of the periventricular white matter with a “capping” pattern was observed adjacent to the frontal horns of the lateral ventricles, suggestive of periventricular leukomalacia (PVL). During the following clinical course, thrombocytopenia and disseminated intravascular coagulation (DIC) developed, prompting discontinuation of metronidazole. The infant received multiple transfusions of platelets, fresh frozen plasma, and albumin, along with antithrombin III infusions. On day 30 of life, six days after transfer from the birth unit to our NICU, the neonate experienced recurrent episodes of desaturation and bradycardia, requiring repeated advanced resuscitation interventions, including chest compressions and intravenous administration of adrenaline. Despite maximal ventilatory support [the lungs showed a diffuse and marked reticulonodular thickening, with alternating multiple cystic-like formations, consistent with a severe bronchopulmonary dysplasia (BPD) pattern, see Figure 2] and aggressive inotropic therapy, the patient's condition evolved in severe hemodynamic instability and multiorgan failure. The infant died 24 h later, at 1 month of age. An autopsy was recommended by the medical team but was declined by the parents. Figure 3 resumes the clinical course of the reported case.

## Discussion

Traumatic pharyngo-esophageal perforation is a rare but recognized complication in preterm neonates, most often related to feeding tube manipulation or airway management (1–6, 15). The structural fragility of the premature esophagus—characterized by connective tissue immaturity, poor vascularization, small lumen diameter, and absence of a protective serosal layer—predisposes ELBW infants to injury even during routine procedures (1–4, 6, 7). Compression against the cervical vertebrae, neck hyperextension during nasogastric or orogastric tube placement, and reflex esophageal contractions further increase the risk of injury (16). In the present case, prematurity and extremely low birth weight were compounded by additional risk factors, including invasive mechanical ventilation, pharmacological treatment for patent ductus arteriosus, and systemic infection (1–7, 15, 17–25). Ibuprofen exposure and barotrauma related to high-pressure ventilation have both been identified in the literature as contributing factors to esophageal or gastrointestinal injury (16, 26). Moreover, ischemic vulnerability and inflammatory states further predispose ELBW infants to gastrointestinal perforations (6, 27–34). The absence of a serosal layer facilitates rapid spread of inflammation, leading to mediastinal and systemic complications, as observed in our patient (4, 6, 7).

The present report is complemented by a focused review on neonatal EP cases published between 2004 and 2025 (35) (Table 1). Compared with previously reported cases, our newborn shared several established risk factors, including extreme prematurity, ELBW, prolonged mechanical ventilation, and repeated orogastric tube manipulation. However, notable differences emerge when considering the clinical course and outcome. While most reported neonatal EP cases describe stabilization and recovery following conservative management, our patient experienced rapid clinical deterioration despite early recognition and appropriate non-surgical treatment. This unfavorable evolution appears primarily related to the cumulative burden of severe prematurity-related comorbidities—including sepsis, evolving BPD, pulmonary hypertension, PVL, and multiorgan dysfunction. While these comorbidities are frequently described in the literature, in our patient the simultaneous presence of multiple interrelated complications, compounded by extreme prematurity and low birth weight, as well as maternal and obstetric risk factors, likely contributed to the fatal outcome despite timely and guideline-consistent management. Furthermore, in our NICU, the standard clinical practice is to replace nasogastric tubes (NGT) every 5–7 days, or earlier if there is malposition, obstruction, or signs of dislodgement. In the present case, the NGT was initially placed within the first 72 h of life to allow minimal enteral feeding with the mother's milk, and then regularly replaced according to protocol. Subsequent imaging, including chest and abdominal x-rays and ultrasonography, was performed during the second and early third week of life; malposition of the tube was noted on day 24 of life, with the tip observed just below the diaphragm in the right hypochondrium. This finding was associated with difficulty in repositioning the tube. Consequently, a short oral tube was placed to allow superficial oropharyngeal suctioning of secretions, avoiding further attempts to insert the NGT and minimizing the risk of additional iatrogenic injury. The initial manifestations of esophageal perforation (EP) are often nonspecific and can involve abdominal distension, hypersalivation, choking, coughing, cyanosis, feeding intolerance, vomiting, and gasping (25). Other clinical signs may be gastrointestinal bleeding, hemoptysis, respiratory distress, apnea, pneumothorax, and pneumonia. Common comorbidities associated with EP are intraventricular hemorrhage (IVH), retinopathy of prematurity (ROP), respiratory distress syndrome (RDS), bronchopulmonary dysplasia (BPD), and acute kidney injury (AKI) (2, 3, 15, 19). In our patient PVL, rarely reported in the literature, was additionally observed. All these conditions are frequently seen in preterm and VLBW neonates, whose physiological immaturity, systemic fragility, and need for intensive cardiorespiratory support involving multiple invasive procedures and hemodynamic stress expose them to a higher inherent risk of such complications (24). However, EP itself can also contribute to the development or worsening of these conditions by triggering systemic inflammation, exacerbating respiratory failure, impairing renal perfusion, and increasing the overall risk of multiorgan dysfunction. Diagnosis is typically confirmed via chest x-ray, showing misplacement or displacement of the nasogastric (NGT) or orogastric (OGT) tube, especially if it enters the pleural space, as occurred in the presented patient. Additional findings such as pneumothorax, pleural effusion, and pneumomediastinum may also be visible, with the former being evident also in the present patient. A lateral view taken simultaneously can help raise diagnostic suspicion. However, 12%–33% of EP cases may appear normal on plain radiographs, and in such cases a water-soluble contrast esophagogram can offer further diagnostic insight. Esophageal atresia must also be considered and excluded in the differential diagnosis, as the absence of contrast in the stomach and small bowel can strongly support the diagnosis of perforation over other potential pathologies (16).

**Table 1:** The table summarizes and compares the clinical characteristics of preterm patients with esophageal perforation (case reports and case series) described in the literature, with the patient reported in the present case report added at the bottom.

Authors	Study design	Cases (n)	GA (weeks and median)	BW(range and median, gr)	Sex	Type of delivery	Maternal factors	Neonatal factors	Etiology and additional risk	Complications and Comorbidities	Diagnostic modalities	Management
									Factors			
Emil (17)	Case report	1	26	900	F	Vaginal	N/S	Preterm	Intubation	RDS, Gastroesophageal reflux	Surgical exploration,	Operative
								ELBW	OGT		Esophagogram,	[Thoracotomy]
											Chest x-ray	
Suryawanshi et al. (18)	Case report	1	27	900	M	Cesarean section	N/S	Preterm, ELBW	OGT	Sepsis, RDS	Esophagogram	Conservative
Hesketh et al. (3)	Case series	7	24–36	450–2,315	M (4)	N/S	Abruptio placentae	Preterm, LBW	OGT, NGT	Sepsis, NEC, RDS, PDA	Chest x-ray,	Conservative (7)
			(25^+6^)	−600	F (3)		Chorioamnionitis	ELBW, LBW	Intubation	PNX, PNM, IVH, PNP, Cardiac arrest	Tube contrast study,	
								IUGR			Esophagoscopy,	
											Esophagogram	
Onwuka et al. (5)	Observational retrospective study	25	24–29 (26^+5^)	540–1,410 (900)	M (13) F (12)	Cesarean section (21) Vaginal (4)	Twinning	Preterm, ELBW, VLBW, twins	OGT, NGT	Sepsis, Pneumonia, PNX, Respiratory failure, Prolonged ventilation, nosocomial infections, NEC, IVH	Esophagogram	Conservative (25)
Yong et al. (20)	Case series	3	23–27	585–995	N/S	Cesarean Section (2)	History of Multiple	Preterm	Intubation	Bacteremia, Cerebral ventriculomegaly	Chest x-ray	Conservative (3)
			−25	−650		Vaginal (1)	Abortions	ELBW	NGT	PNP, Subependymal cyst,		
										RDS, ROP, IVH, PNX		
Lithoxopoulou et al. (21)	Case report	1	28	1,100	M	Cesarean section	Chorioamnionitis, PROM	Preterm, VLBW	NGT (posterior cervical esophageal injury)	Sepsis, RDS, thrombocytopenia	Esophagogram	Conservative
Adel et al. (4)	Observational cross-sectional study	15	27–34	780–1,800	am	Cesarean Section (12)	Hypertension, PROM	Preterm, LBW	Intubation	Pneumonia, Sepsis, Tricuspid Regurgitation,	Esophagogram	Conservative (15)
			−30	−1,100	F (7)	Vaginal (3)	Decollement, GDM	ELBW, IUGR	OGT	LVH, RDS, PDA, ASD, VSD, PNX		
							Placenta previa	VLBW				
							Preeclampsia					
Mikołajczak et al. (22)	Multicenter retrospective study	10	23–35	430–1,100	M (5)	Cesarean Section (4)	Chorioamnionitis	ELBW, VLBW	OGT or NGT	BPD, PE, Milky PE, Ileum perforation, PDA,	Chest-x-Ray	Conservative (8)
			(24^+3^)	−640	F (5)	Vaginal (6)		Preterm, SGA	Intubation	Airl leak [PNX, PNM], Sepsis, Renal failure Fungemia, Pneumonia,		Surgical (2)
								IUGR		Cardiorespiratory decompression, IVH,		
										Peritonitis, MOF, RDS, NEC		
Sorensen et al. (23)	Multicenter retrospective study	8	23^+4^–39	511–3,500	M (7)	N/S	N/S	Preterm, LBW	OGT or NGT	Acute on Chronic Renal Failure, Septic Shock, Pleural effusion, BPD, IVH	Chest x-ray	Conservative (8)
			(26^+4^)	−636	F (1)			ELBW	Intubation	Mediastinal abscess, RDS, PNX		
Aljadaan et al. (24)	Case series	7	22^+5^–27	460–840	M (3)	N/S	N/S	Preterm, ELBW, IUGR Ambiguous genitalia	Intubation	Periventricular leukomalacia, PDA, NEC, Sepsis, Left atrium and ventricle dilatation, BPD, PNX,	Surgical exploration	Conservative (7)
			(24^+4^)	−576	F (4)				OGT	Lung collapse, Renal failure, ASD, IVH, RDS,	Esophagogram	
										PNM, TPN-related liver disease	Chest x-ray	
Eguchi et al. (25)	Observational retrospective study	6	23^+5^–28^+6^	630–1,232	M (4)	N/S	N/S	Preterm, ELBW	OGT or NGT	Mediastinitis,	Laryngoscopy	Conservative (6)
			(27^+1^)	−823	F (2)			VLBW, Triplet	Intubation	PNX, PDA, RDS, IVH	Esophagogram	
								SGA			Chest x-ray	
Our patient	Case report	1	27	480	M	Cesarean section	Preeclampsia	Preterm, ELBW, IUGR, SGA	Intubation, OGT	PNM, PNX, RDS, PDA, HCM, BPD, sepsis, pulmonary hypertension	Ultrasound,	Conservative
											Chest x-Ray	

ASD, Atrial Septal Defect; BPD, Bronchopulmonary Dysplasia; GDM, Gestational Diabetes Mellitus; HCM, hypertrophic cardiomyopathy; LBW, Low Birth Weight; LVH, Left ventricular hypertrophy; MOF, Multi-organ Failure; NEC, Necrotizing Enterocolitis; N/S, Not specified; PE, Pleural effusion; PNM, Pneumomediastinum; PNP, Pneumoperitoneum; PNX, Pneumothorax; PROM, Premature Rupture of Membranes; PVL, Periventricular Leukomalacia; RDS, Respiratory Distress Syndrome; SGA, Small for Gestational Age; TPN, Total parenteral nutrition VSD, Ventricular Septal Defect.

In the presented case, most of the typical risk factors (i.e., preeclampsia), clinical manifestations, imaging findings, and conditions associated with EP were present, including abdominal distension, difficulty in repositioning the tube, RDS, BPD, sepsis, pneumothorax—initially observed on plain x-ray—and later, mediastinal emphysema clearly detected on cardiac US. Management of iatrogenic EP in neonates depends on the severity and presentation of each case, but early diagnosis is crucial for initiating conservative management. Typically, it involves removing the misplaced nasogastric or orogastric tube, withholding oral feeding (*nil per os*), providing adequate fluid resuscitation, administering broad-spectrum antibiotics to cover anaerobic and enteric bacteria, offering parenteral nutrition, and supporting respiratory function or managing comorbidities (4, 23). Surgical intervention is seldom required, and is reserved for cases of pneumothorax or mediastinal emphysema unresponsive to drainage, complicated mediastinitis, abscess formation, significant extravasation on contrast studies, or failure of conservative management. Surgical treatment in other clinical scenarios has not been shown to improve survival outcomes (19). After surgery, enteral nutrition is typically provided through gastrostomy. In patients conservatively managed, total parenteral nutrition for at least 10 days should be performed, as oral feeding should not be resumed before this period. In critically ill patients with prolonged recovery, enteral nutrition may be resumed via gastrostomy, and oral feeding may be delayed further if the EP has not fully healed (23). Once healing is complete, NGT/OGT placement can be safely performed under fluoroscopic guidance using a radiopaque guide and/or a water-soluble contrast agent. If the contrast remains confined to the esophagus and stomach, the perforation is considered resolved, allowing for further tube advancement and reintroduction of enteral nutrition (36). In recent years, endoscopic approaches have also been described as potential therapeutic options in selected cases of neonatal esophageal perforation (37). Endoscopic evaluation allows direct visualization of the lesion and, in carefully chosen stable patients, may support minimally invasive interventions such as endoscopic clipping, fibrin glue application, or temporary stent placement (38). However, experience with these techniques in neonates—particularly in ELBW infants—remains limited and is largely confined to isolated case reports and small series (37–39). The feasibility and safety of endoscopic treatment rely on multiple factors, including patient stability, size and location of the perforation, availability of specialized expertise, and the risks associated with anesthesia and airway manipulation. Consequently, endoscopic management should be considered only in highly selected cases and performed in specialized centers (37). In the present case, despite the theoretical availability of additional therapeutic options, including endoscopic ones, the infant's extreme clinical instability rendered such intervention inappropriate. Conservative management was therefore deemed the most suitable strategy, in line with the principles of proportionality of care and minimization of further iatrogenic harm as well as literature evidence, including pneumothorax drainage which was also required. Unfortunately, the neonate's deteriorating clinical condition precluded the further placement of NGT/OGT, as septic and respiratory complications associated with EP ultimately led to a fatal outcome.

Measures to reduce the risk of EP include careful procedural technique and appropriate choice of feeding tubes. Key preventive aspects involve gentle insertion with minimal force and adequate lubrication, accurate tube length estimation [e.g., NEMU (Nose–Ear–Mid–Xiphoid–Umbilicus) or NEX (Nose–Earlobe–Xiphoid) methods], verification of tube placement via radiography or ultrasound before feeding, limiting repeated insertion attempts in unstable or extremely preterm infants, and regular staff training, including simulation-based practice, to ensure safe handling of fragile neonates (20, 34, 40–42). Specifically, applying gentle pressure during insertion to verify correct placement and prolonging intervals between tube replacements were both considered and enforced by the neonatologists in the present case. Moreover, several studies suggest that using slender silicone tubes, which are more flexible and less stiff than polyvinyl ones (those used in our patient), increases the likelihood of correct tube placement (43, 44). Additionally, manufacturers recommend improving the texture of gastric tubes by soaking them in warm water and gently manipulating them to improve softness, as well as applying adequate lubrication (20). In our NICU, silicone devices are not available, so we rely on additional precautions such as moistening the tube with warm water, as performed in our newborn, to reduce the risk of tissue injury, particularly high for VLBW or extremely preterm neonates. Although NGT/OGT insertion has traditionally been regarded as the primary precipitating event, endotracheal intubation represents an equally relevant potential cause and warrants comparable attention. Because both procedures are commonly performed within the first hours of life, distinguishing which intervention directly led to the perforation may be challenging. Preventive strategies should therefore address also airway procedures. Specifically, the adoption of less traumatic intubation devices, such as plastic-blade video laryngoscopes (e.g., Parker Neonatal Video Laryngoscope), may help further mitigate the danger of iatrogenic injury (45). In our case, however, it appears plausible that the orogastric tube was the primary cause of EP rather than the endotracheal tube, as clinical deterioration occurred immediately following an attempt of OGT repositioning.

It is crucial to maintain a high index of suspicion for misplacement, particularly in high-risk patients or when difficulties arise during NGT/OGT positioning. Therefore, in addition to training specialized personnel, it may also be beneficial to assess the correct tube location using US. US-guided procedures are gaining more and more applications in many different fields of pediatric care (46, 47), and among these, US-guided NGT/OGT placement is emerging as an effective, efficient, and reliable technique in neonatal care. This method offers several advantages, including widespread availability in NICUs, cost-effectiveness, and reduced radiation exposure compared to traditional radiographic approaches. It provides real-time imaging of tube progression without the use of radiation, and has demonstrated a high diagnostic accuracy (92.2%) in assessing NGT/OGT placement in neonates (42). However, US-based verification does have limitations, such as reduced visibility in cases of hepatomegaly, excessive patient movement, interference from intestinal gas, and reliance on operator expertise. In our case, the clinical deterioration and difficulties encountered during OGT repositioning initially raised the suspicion of esophageal perforation (EP). This was later supported through bedside US and, subsequently, chest and abdominal x-rays, which ultimately revealed tube misplacement and a right-sided PNX, despite the initial absence of free abdominal air or mediastinal emphysema. Subsequent imaging, including echocardiography, and chest x-Rays revealed signs of mediastinal emphysema and of reducing pneumothorax. These findings were consistent with the EP diagnosis, and further allowed monitoring of the evolution of perforation-related complications. Therefore, additional invasive diagnostic procedures, such as contrast esophagogram and/or endoscopy, were deemed unnecessary. These investigations may, however, be indicated in more stable patients when the diagnosis remains uncertain due to inconclusive first-level imaging findings. Although radiography remains the gold standard for confirming tube position, its routine use should be carefully considered due to the potential risks associated with cumulative radiation exposure. In our experience, the number of radiographic examinations was effectively balanced by ultrasound, especially for post-acute evaluations.

Although the mechanical complication—the esophageal perforation—was successfully contained through conservative treatment, the overall outcome of our patient was dictated by the extreme immaturity and subsequent septic deterioration. This case underscores the intrinsic vulnerability of ELBW infants and the fine balance between necessary invasive procedures and the risk of iatrogenic injury. It also emphasizes the diagnostic value of bedside US in recognizing tube misplacement early and guiding subsequent management while avoiding repeated radiation exposure. Beyond the technical aspects, this experience raised important ethical and communicative challenges. The medical team was required to navigate complex discussions with the parents regarding prognosis, potential complications, and limits of intensive therapy in an infant at the threshold of viability. Transparent communication, shared decision-making, and compassionate accompaniment proved essential elements of care. This ethically guided and family-centered approach may also have contributed to the couple's decision not to proceed with an anatomopathological examination of their child after death.

### Ethical and communication perspectives

Beyond its clinical aspects, neonatal EP often raises profound ethical and communicative challenges, particularly in extremely preterm infants at the threshold of viability (8). The sudden onset of a severe iatrogenic complication may provoke distress and guilt among caregivers, as well as anxiety and loss of trust among parents. Transparent, compassionate communication is therefore essential. Parents must be informed promptly, with explanations that balance honesty and reassurance (48). The clinician's responsibility extends beyond disclosing the event to supporting parental understanding and preserving the therapeutic alliance. Multidisciplinary debriefings can help align the medical team and ensure coherence in communication (8, 34, 40, 41, 49–56). From an ethical standpoint, EP exemplifies the broader tension between beneficence and non-maleficence in neonatal care: every intervention intended to sustain life carries an inherent risk of harm. Decisions regarding escalation or withdrawal of care in the presence of severe complications must be guided by the infant's best interests, proportionality of treatment, and shared decision-making with the family.

In critically ill neonates, ethical decision-making is further complicated when considering additional diagnostic or therapeutic procedures, such as contrast studies or endoscopic interventions. Although these approaches may be appropriate in selected stable patients, their risks may outweigh potential benefits in unstable ELBW infants. In our case, avoiding further invasive procedures was consistent with the ethical principle of proportionality, prioritizing comfort and minimizing additional harm in a context of limited therapeutic reversibility.

## Conclusions

This case illustrates the potentially life-threatening impact of esophageal perforation in ELBW infants, even when promptly recognized and managed according to current conservative standards. Our experience suggests that, in the most vulnerable neonates, outcomes are often dictated not by the perforation itself but by the cumulative burden of prematurity-related comorbidities and systemic instability. Bedside US emerged as a valuable diagnostic adjunct, facilitating early detection and follow-up while reducing radiation exposure. Beyond clinical management, this case underscores the ethical complexity of neonatal intensive care when iatrogenic complications occur in infants at the limits of viability. Transparent communication, shared decision-making, and proportionality of care remain essential to align treatment with the infant's best interests and the family's values. Continued reporting of such cases contributes to improving awareness, refining preventive strategies, and strengthening ethical reflection in neonatal practice.
